# Personalized evidence of treatment effects for the practice of personalized medicine: a new model of care

**DOI:** 10.1186/1472-6963-14-S2-P17

**Published:** 2014-07-07

**Authors:** Jean-Paul Collet, William McKellin, Sravan Jaggumantri, Niranjan Kissoon

**Affiliations:** 1 Department of Pediatrics, University of British Columbia, Vancouver, BC, Canada; 2 Child and Family Research Institute, Vancouver, BC, Canada; 3 Department of Anthropology, University of British Columbiac, Vancouver, BC, Canada; 4 British Columbia Children’s Hospital, Vancouver, BC, Canada

## 

Personalized medicine, based on individuals’ genomes or proteomes challenges conventional clinical diagnostic categories and increases the number of “rare conditions” which may benefit from personalized treatments. Current drug evaluation, based on diagnostic categories ignores the large range of phenotypic expressions; the considerable variation in drug responses by individuals creates legitimate concerns regarding the efficacy and safety of many drugs for patients. Furthermore, when diseases affect individuals’ daily activities, standardized population-based assessment tools lack the capacity to reliably reflect a drug’s effects on outcomes most relevant to the patient’s own everyday needs and perception of wellness - the individual patient’s Personal Outcomes of Specific Interest (“POSI”). The uniqueness of individual’s POSI challenges the classic phase 3 trials to assess drug effects; conversely, the validity of evaluating drug effects with standard tools – surrogate markers of a true experience – is questionable.

It is therefore essential to develop a new approach to carefully assess the impact of these drugs on the outcomes that are most important for patients while also assessing the bio-physiological effectiveness.

In 2010, we developed a personalized medicine strategy using a systematic consultative model of decision-making that incorporates individual patients/families’ insights about the impact of drugs on everyday activities and their preferred outcomes into the treatment evaluation and decision making process; we also developed with UBC School of Engineering the “Wellness Tracker” (WT) software that enables patients/families to track signs and symptoms on a daily basis, and share this information with caregivers (http://wellnesstracker.org), to guide therapeutics and decisions. WT can be used in n-of-1 clinical trials that are the best design for assessing drug effectiveness and side effects at individual and population levels.

The personal evaluation model (PEM) includes 4 steps to: (i) identify POSI with the patient/family in consultation with the physician; (ii) determine which drug effects would make the treatment useful; (iii) assess changes in POSI over time and scale this change using the goal attainment scaling (GAS) technique; (iv) share this information to the discussion with care providers to make the most appropriate decisions regarding drug usage and adherence considering utility scorings from the different parties.

This approach also provides important data for policy makers to evaluate reimbursement for expensive treatments. Finally, it can also be used to study off-label applications of previously approved drugs, herbal products and dietary supplements; most of which are never evaluated rigorously. Presentation illustrates use of Personalized Evaluation Model in practice.

